# *SOAT1* Nonsense Variant in a Cat With Sebaceous Gland Dysplasia

**DOI:** 10.1002/age.70180

**Published:** 2026-08-10

**Authors:** Lia Kammermann, Joanne K. Mansell, Barbara G. McMahill, Verena K. Affolter, Vidhya Jagannathan, Tosso Leeb

**Affiliations:** ^1^ Institute of Genetics, Vetsuisse Faculty University of Bern Bern Switzerland; ^2^ Dermatopathology Specialty Service, College of Veterinary Medicine and Biomedical Sciences Texas A&M University College Station Texas USA; ^3^ Pathology Services IDEXX Reference Laboratories Inc. Lander Wyoming USA; ^4^ Department of Pathology, Microbiology, Immunology, School of Veterinary Medicine University California Davis Davis California USA

**Keywords:** animal model, dermatology, *Felis catus*, precision medicine, veterinary medicine

## Abstract

We investigated a random‐bred cat with sebaceous gland dysplasia. The cat presented with hypotrichosis and dark crusts on the skin. Histopathologically, sebaceous glands were enlarged and had an abnormal morphology with an increased number of undifferentiated reserve cells. Whole genome sequence analysis of the affected cat and comparison to 106 genomes of genetically diverse cats revealed a private homozygous nonsense variant in the functional candidate gene *SOAT1* encoding sterol O‐acyltransferase 1, XM_011291017.4:c.1221G>A or XP_011289319.1:p.(Trp407*). Based on the known role of *SOAT1* in sebaceous gland function, the identified variant most likely represents the cause for the phenotype. To the best of our knowledge, we report the second pathogenic *SOAT1* variant in cats. These results enable diagnostic genetic testing should any new cases come under veterinary care, detection of unaffected carrier animals, and strengthen the gene‐phenotype relationship between *SOAT1* and sebaceous gland dysplasias.

1

Sebaceous glands are acinar holocrine glands in the skin associated with hair follicles as part of the pilosebaceous unit. Their lipid‐rich secretion, sebum, is composed of disintegrated sebocytes which release their lipid‐rich contents, including triglycerides, cholesterol, cholesterol esters, free fatty acids, wax esters, and squalene. Sebum greases hair shafts, assists removal of keratin from hair follicle, can influence follicular cycle, lubricates the skin, and plays an important role in skin integrity (Niemann and Horsley 2012; Geueke and Niemann 2021; Mosca et al. 2025; Shamloul and Khachemoune 2020). Similarly, as modified sebaceous glands, Meibomian glands in the eyelids also produce cholesterol esters, an important component of the tear film (Butovich 2017).

Cats with sebaceous gland dysplasia, characterized by morphologically abnormal sebaceous glands, present with progressive hypotrichosis with variable scaling and crusting (Yager et al. 2012). In two cats with an autosomal recessive form of sebaceous gland dysplasia, a missense variant in the *SOAT1* gene encoding sterol O‐acyltransferase 1 was identified as the underlying cause (Kiener et al. 2023; OMIA:002669–9685). *Soat1* ^*−/−*^ mice also display abnormalities in the hair and tear film composition (Butovich et al. 2021; Potter et al. 2015; Wu et al. 2010). Sterol O‐acyltransferase 1 catalyzes the formation of cholesterol esters and *SOAT1* is currently the only known functional candidate gene for the phenotype of sebaceous gland dysplasia. Genetic heterogeneity is considered likely, as no pathogenic variants in *SOAT1* were found in other cats with sebaceous gland dysplasia at that time (Kiener et al. 2023).

In this study, we investigated a new case of feline sebaceous gland dysplasia. A five‐and‐a‐half‐month‐old, random‐bred shorthaired feral cat was initially presented with bilateral symmetrical scaling with black debris, hypotrichosis and hair clumping on the bridge of the nose. The cat was unthrifty, thin and pot‐bellied. Ancillary testing, including FeLV, FIV, coprology and dermatophyte culture were negative. For the next 3 months the skin lesions progressively worsened to affect the whole muzzle, periocular locations, ears and all four legs (Figure 1a). There was no response to empirical antibiotic therapy. At that time skin biopsy was pursued. Four skin biopsies from right hind leg, left hind leg, chin and right side muzzle were submitted for histopathological evaluation and revealed similar findings. Sebaceous glands were variably enlarged with abnormal morphology (Figure 1b). Sebaceous glands were characterized by single or tiny clusters of sebocytes surrounded by increased numbers of reserve cells. Occasional apoptotic sebocytes were present in these clusters (Figure 1b). Hair follicles were cycling but follicular dysplasia was observed with wavy contours and misshapen walls. The epidermis varied from normal thickness to markedly hyperplastic, covered by diffuse marked orthokeratosis with multifocal serocellular crusts. With these findings, a diagnosis of sebaceous gland dysplasia was made.

**FIGURE 1:**
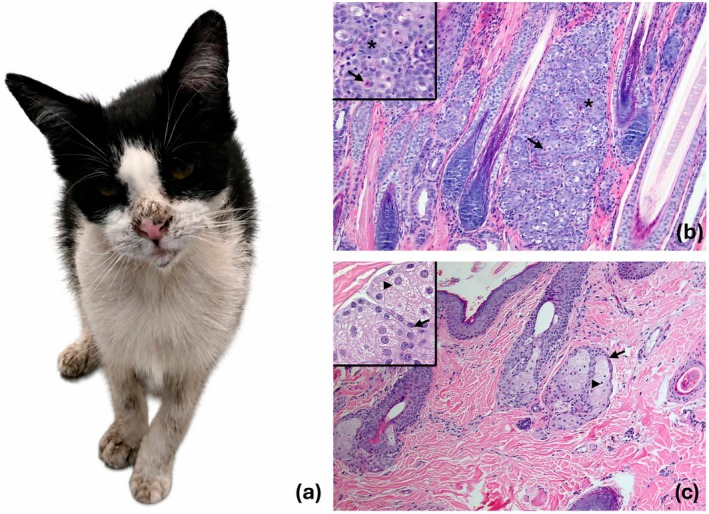
Clinical and histopathological phenotype. (a) Bilateral symmetrical scaling and black debris with hypotrichosis affecting face, legs and paws. (b) Abnormal sebaceous gland architecture and differentiation in haired skin from proximal hind leg. H&E 100×. There is an increased number of reserve cells (undifferentiated sebocytes, marked by an asterisk) and apoptotic cells (shrunken eosinophilic cells, arrow). Inset, top left corner, 400×. (c) Age matched control, haired skin: Note expected architecture with normal maturation from reserve cell (arrow) to sebocyte (arrowhead). Even though the body location of the control sample was unfortunately not known, the histologic appearance of sebaceous glands is expected to remain the same in all body locations. H&E 100×. Inset, top left corner, 400×.

For genetic investigations, genomic DNA was isolated from an EDTA blood sample of the affected cat, which was processed within the VET_GEN_BERN collection of the Vetsuisse Biobank. Whole genome sequencing was performed at 23× coverage on an Illumina Novaseq 6000 instrument. Read mapping to the F.catus_Fca126_mat1.0 reference genome assembly and variant calling were performed as described (Jagannathan et al. 2019).

We filtered for private variants in the affected cat by comparing the data to whole genome sequence data from 106 genetically diverse control genomes (Table S1). The analysis identified 686 heterozygous and 194 homozygous private protein‐changing variants (Table S2). Only one of them, a homozygous single nucleotide substitution, was located in *SOAT1*, the top candidate gene for the phenotype. This variant, ChrF1:NC_058384.1:g.20904899G>A or XM_011291017.4:c.1221G>A was a nonsense variant, predicted to truncate 123 codons (22%) of the wildtype open reading frame, XP_011289319.1:p.(Trp407*) including the coding information for the last three transmembrane domains. Wildtype sterol O‐acyltransferase 1 has 9 transmembrane domains and is located in the membrane of the endoplasmic reticulum (Long et al. 2020; Qian et al. 2020). The nonsense variant is therefore likely to completely disrupt gene function. We did not experimentally investigate whether a mutant truncated SOAT1 protein is actually expressed.

Integrated clinical and histopathology findings confirm feline sebaceous gland dysplasia for this patient. Furthermore, the early onset aligns with previous studies (Yager et al. 2012; Kiener et al. 2023). Scaling, hypotrichosis and alopecia are the dominant clinical signs, while on histopathology abnormal sebaceous gland morphology and differentiation are the prominent pattern, which is also in accordance with earlier observations. The findings in this cat strengthen the identification of *SOAT1* as a candidate gene for sebaceous gland dysplasia as previously described by Kiener et al. (2023). At the time of preparing this manuscript, the patient has reached an age of 9 years and is doing very well, with good appetite and energy level. The skin lesions are purely cosmetic and are managed by the owner with bathing.

We acknowledge several limitations of our study. The investigated cat was a single, non‐pedigreed individual. We did not have access to samples from relatives to confirm correct genotype–phenotype co‐segregation. Applying the ACMG/AMP criteria for the interpretation of sequence variants in human diagnostics (Richards et al. 2015) or the AVCG v2 criteria (Desmet et al. 2026), we classified the variant as pathogenic (Table S3).

In conclusion, we consider the *SOAT1*:c.1221G>A nonsense variant to be the most likely causative variant for the sebaceous gland dysplasia in the studied cat. To our knowledge, this is the second reported pathogenic *SOAT1* variant. The findings enable diagnostic genetic testing should any new cases come under veterinary care, detection of unaffected carrier animals, and strengthen the gene‐phenotype relationship between *SOAT1* and sebaceous gland dysplasia.

## Author Contributions

**Lia Kammermann:** investigation, writing – original draft, writing – review and editing. **Vidhya Jagannathan:** data curation, writing – review and editing. **Tosso Leeb:** conceptualization, writing – original draft, writing – review and editing, supervision. **Joanne K. Mansell:** investigation, writing – review and editing. **Barbara G. McMahill:** investigation, visualization, writing – original draft, writing – review and editing, conceptualization. **Verena K. Affolter:** investigation, writing – review and editing.

## Ethics Statement

The cat in this study was privately owned and skin biopsies and the EDTA blood sample were collected with the consent of its owner. Sampling of the affected cat by the attending veterinarian was performed for diagnostic reasons and did not constitute an animal experiment in the legal sense.

## Conflicts of Interest

The authors declare no conflicts of interest.

## Supporting information


**Table S1:** Accession numbers of 111 cat genome sequences. The affected cat is highlighted in red. Four cats with the same phenotype were excluded (highlighted in blue). The other 106 genome sequences were used as controls in variant filtering.

**Table S2:** Private variants in the sequenced cat affected with sebaceous gland dysplasia. Private variants were obtained by filtering out all variants that were present at least once in the 106 control genomes. This resulted in 152 506 heterozygous private variants for the affected cat. Please note that variants are listed multiple times, if they have predicted effects on more than one transcript.

**Table S3:** Classification of the XM_011291017.4:c.1221G>A variant according to the human ACMG/AMP and AVCG v2 criteria.

## Data Availability

All data are freely available. Accessions for the whole genome sequence data are given in Table S1.
